# Interactions between cranberries and fungi: the proposed function of organic acids in virulence suppression of fruit rot fungi

**DOI:** 10.3389/fmicb.2015.00835

**Published:** 2015-08-14

**Authors:** Mariusz Tadych, Nicholi Vorsa, Yifei Wang, Marshall S. Bergen, Jennifer Johnson-Cicalese, James J. Polashock, James F. White

**Affiliations:** ^1^Department of Plant Biology and Pathology, Rutgers UniversityNew Brunswick, NJ, USA; ^2^Philip E. Marucci Center for Blueberry and Cranberry Research and Extension, Rutgers UniversityChatsworth, NJ, USA; ^3^Genetic Improvement of Fruits and Vegetables Laboratory, United States Department of Agriculture-Agriculture Research Service, Philip E. Marucci Center for Blueberry and Cranberry Research and ExtensionChatsworth, NJ, USA

**Keywords:** benzoic acid, bioactivity, cranberry fruit rot disease, pathogenicity, quinic acid, reactive oxygen species, resistance, *Vaccinium*

## Abstract

Cranberry fruit are a rich source of bioactive compounds that may function as constitutive or inducible barriers against rot-inducing fungi. The content and composition of these compounds change as the season progresses. Several necrotrophic fungi cause cranberry fruit rot disease complex. These fungi remain mostly asymptomatic until the fruit begins to mature in late August. Temporal fluctuations and quantitative differences in selected organic acid profiles between fruit of six cranberry genotypes during the growing season were observed. The concentration of benzoic acid in fruit increased while quinic acid decreased throughout fruit development. In general, more rot-resistant genotypes (RR) showed higher levels of benzoic acid early in fruit development and more gradual decline in quinic acid levels than that observed in the more rot-susceptible genotypes. We evaluated antifungal activities of selected cranberry constituents and found that most bioactive compounds either had no effects or stimulated growth or reactive oxygen species (ROS) secretion of four tested cranberry fruit rot fungi, while benzoic acid and quinic acid reduced growth and suppressed secretion of ROS by these fungi. We propose that variation in the levels of ROS suppressive compounds, such as benzoic and quinic acids, may influence virulence by the fruit rot fungi. Selection for crops that maintain high levels of virulence suppressive compounds could yield new disease resistant varieties. This could represent a new strategy for control of disease caused by necrotrophic pathogens that exhibit a latent or endophytic phase.

## Introduction

Many pathogens possess a latent phase where they grow within tissues of hosts without causing harm or resulting in expression of disease symptoms (Luo and Michailides, 2001; Sauer et al., 2002; Vega et al., 2010; O'Connell et al., 2012; Tadych et al., 2012; Delaye et al., 2013). It is only after this period of latent development that disease expression may become evident. Our previous study (Tadych et al., 2012) suggested that the majority of cranberry fruit rot necrotrophic fungi possess a latent phase in which they grow inside cranberry fruit asymptomatically. Fruit rot fungi may coexist in developing ovaries as endophytes or they may exist alone in ovaries. After this phase of symptomless development, usually shortly before or at fruit maturation, disease expression in the form of rot may become evident.

Fungal diseases, particularly the cranberry fruit rot disease complex, have been serious problems limiting fruit production from the beginning of commercial cultivation of American cranberry (*Vaccinium macrocarpon* Aiton) (Halsted, 1889; Stevens, 1924; Shear et al., 1931; Oudemans et al., 1998; Tadych et al., 2012). Among the most common fungi causing cranberry fruit rot disease are *Coleophoma empetri* (Rostr.) Petr., *Colletotrichum acutatum* J. H. Simmonds, *Colletotrichum gloeosporioides* (Penz.) Penz. & Sacc., *Fusicoccum putrefaciens* Shear, *Phomopsis vaccinii* Shear, N. E. Stevens & H. F. Bain, *Phyllosticta vaccinii* Earle and *Physalospora vaccinii* (Shear) Arx & E. Müll (Oudemans et al., 1998; Polashock et al., 2009; Tadych et al., 2012).

Defensive mechanisms against pathogens in many animals and plants involve the direct action of reactive oxygen species (ROS), such as superoxide (O^−^_2_), hydroxyl radical (OH^•^), and hydrogen peroxide (H_2_O_2_) (Foyer and Harbinson, 1994; Wu et al., 1997; Missall et al., 2004; Silar, 2005). It has been shown that ROS are generated as anti-pathogen agents and as warning signals to adjacent host cells, triggering other host defensive reactions (Lamb and Dixon, 1997; Wojtaszek, 1997). Pathogens often trigger an increase in ROS called “oxidative burst,” which results in the accumulation of ROS in tissues of the plant proximal to the pathogen (Apel and Hirt, 2004). The accumulation of ROS may cause damage to cells by peroxidizing lipids and disrupting structural proteins, enzymes, and nucleic acids, and may subsequently lead to cell death (Apel and Hirt, 2004).

Previous research has associated ROS secretion by fungal necrotrophs with induction of cell death and necrosis in host tissues (Álvarez-Loayza et al., 2011; Heller and Tudzynski, 2011). The linkage between fungal ROS secretion and initiation of the hypersensitive response in host plant tissues provides a target for identification of natural plant constituents that will prolong the non-destructive latent phase of the cranberry rot fungi.

Many bioactive compounds can function as constitutive or inducible barriers against microbial pathogens, and bioactive compound composition can change in response to microbial attack (Dixon and Paiva, 1995; Grayer and Kokubun, 2001; Miranda et al., 2007; Carlsen et al., 2008; Koskimäki et al., 2009; White and Torres, 2010; Oszmiañski and Wojdył, 2014). Cranberry fruit are known to be rich sources of nutrients and bioactive compounds, including phenolics, flavonoids, sugars, organic acid, etc., (Fellers and Esselen, 1955; Schmid, 1977; Coppola et al., 1978; Mäkinen and Söderling, 1980; Hong and Wrolstad, 1986; Zuo et al., 2002; Zheng and Wang, 2003; Cunningham et al., 2004; Shahidi and Naczk, 2004; Vvedenskaya et al., 2004; Singh et al., 2009; Neto and Vinson, 2011), any of which could have activity against rot-inducing fungi (Marwan and Nagel, 1986a,b; Cushnie and Lamb, 2005). Previous research suggests that fungi that cause cranberry fruit rot disease colonize surface layers of cranberry ovaries early in flower development (Zuckerman, 1958; Tadych et al., 2012) and induce disease in mature fruit tissues possibly by secretion of ROS into fruit, resulting in a cascade of events in fruit tissues that leads to cell death and fruit rot. According to this model, suppression of growth and ROS secretion by fungi will result in suppression of rot disease. We hypothesize that fruit rot resistant selections of cranberry are resistant to rot due to organic acid constituents that enable them to suppress growth and ROS production by cranberry fruit rot fungi. We further hypothesize that levels of organic acids may change as fruit mature, leading to a release of ROS suppression and increase in fungal growth and disease incidence in fruit. Objectives for this study were: (1) to identify naturally occurring chemicals in cranberry fruit that suppress growth of cranberry fruit rot fungi, (2) to determine whether secretion of ROS by these fruit pathogens could be stimulated or inhibited by these cranberry constituents, (3) to investigate the organic acid profiles in the fruit of rot-resistant and rot-susceptible cranberry genotypes at intervals throughout fruit maturation that may coincide with fruit rot occurrence.

## Materials and methods

### Reference compounds and other chemicals

Agarose (A6013), L-Alanine (A7627), Benzoic acid (242381), 3,3′-diaminobenzidine (D5905), Folic acid (F7876), Formic acid (F0507), D-(–)-Fructose (F0127), D-(+)-Glucose (BDH0230), Glycine (G7126), Horseradish peroxidase (P6782), DL-Malic acid (M0875), D-Mannitol (M4125), N-Z-Soy® Peptone (P1265), Pectin from apple (P8471), Phosphoric acid, D-(–)-Quinic acid (138622), Starch from rice (S7260), and Sucrose (50389) were purchased from Sigma-Aldrich Chemical Co. (St. Louis, MO). Acetonitrile (AX0145) was obtained from EMD Millipore (Billercia, MA), Citric acid anhydrous (A940) from Fisher Scientific (Fair Lawn, NJ), Sorbitol (V045-07) from J. T. Baker – Mallinckrodt Baker, Inc. (Phillipsburg, NJ) and Proflo Premium Quality Cottonseed-derived Protein Nutrient (069061) from Trades Protein, Southern Cotton Oil Company (Memphis, TN). All solvents were of HPLC grade and water was of Milli-Q quality (Millipore Corp., Bedford, MA).

### Fungal and plant material used

Cranberry fruit rot fungi used in this study were collected from infected cranberry ovaries in 2009 at the Philip E. Marucci Center for Blueberry and Cranberry Research and Extension of Rutgers University located in Chatsworth, New Jersey (39°42′50.75″N, 74°30′33.07″W; altitude 12 m) as described by Tadych et al. (2012) and stored in −80°C until this study. After removing from freezer the fungi were regrown first in potato dextrose broth (PDB) and then re-cultured on potato dextrose agar (PDA) at room temperature (22°C) for 10 days.

Fruit of the American cranberry used in the present study were represented by four rot-resistant, i.e., ‘US88-1’, ‘US88-30’, ‘US88-79’, ‘US89-3’, and two rot-susceptible, i.e., ‘Mullica Queen’ (MQ) and ‘Stevens’ (ST), genotypes growing at the Philip E. Marucci Center for Blueberry and Cranberry Research and Extension at Rutgers University (Vorsa and Johnson-Cicalese, 2012). The plants were planted in randomized block design with 54 rows and 12 columns with one genotype per plot established in 2009. The experimental plots from which the plant material was collected were not fungicide treated and were cultivated in an identical way during the 2012 and 2013 growing season.

On July 11, 2012 green cranberry fruit were collected from US88-79 and ST genotypes, at the phenological stage of “small fruit” (Brown and McNeil, 2006) and transferred to the laboratory. The fruit were ground in liquid nitrogen and added to the basal medium (0.5% agarose + 0.5% glucose) to reach a final concentration of 10% fruit tissue in the medium (Table 1) and tested for the impact on growth and ROS secretion of cranberry fruit rot fungi. In addition, in order to test the influence of sterilization on antimicrobial activity of the fruit, in one case raw fruit tissue was added to an already autoclaved medium [raw rot-resistant genotype green berries – raw resistant (RR); raw rot-susceptible genotype green berries – raw susceptible (RS)], and in another case the fruit tissue was added to a medium before medium autoclaving [autoclaved rot-resistant genotype green berries – autoclaved resistant (AR); autoclaved rot-susceptible genotype green berries – autoclaved susceptible (AS)].

**Table 1:** **Cranberry constituents screened for inhibitory effects on fungal growth and hydrogen peroxide production by selected cranberry fruit rot fungi**.

**Compound Group**	**Compound**	**Concentration (%)**	
		Reported^*^	Used^**^
Amino acids	Alanine	0.1	0.1
	Glycine	0.1	0.1
Organic acids	Benzoic acid	0.02–0.065	0.1
	Citric acid	0.5–1.3	0.02
	Folic acid	N/A	0.1
	Malic acid	0.26–1.14	0.02
	Quinic acid	0.5–1.62	1.0
Reducing sugars	Fructose	0.9–1.5	1.0
	Glucose	3.7–5.0	4.0
Disaccharide	Sucrose	0.215–0.275	0.5
Polysaccharide	Pectin	1.2	1.0
	Starch	0.8–2.6	1.5
Sugar alcohols	Mannitol	0.05	0.5
	Sorbitol	0.05	0.5
Protein	Peptone	N/A	0.1
	Protein	(0.1)/N/A	0.1
**GREEN CRANBERRY FRUIT**			
Rot-resistant genotype	Raw tissues	N/A	10.0
	Autoclaved tissues	N/A	10.0
Rot-susceptible genotype	Raw tissues	N/A	10.0
	Autoclaved tissues	N/A	10.0

* *Concentration of a particular compound found in cranberry fruit (as previously reported: Fellers and Esselen, 1955; Schmid, 1977; Coppola et al., 1978; Mäkinen and Söderling, 1980; Hong and Wrolstad, 1986; Zuo et al., 2002; Zheng and Wang, 2003; Cunningham et al., 2004; Shahidi and Naczk, 2004; Vvedenskaya et al., 2004; Singh et al., 2009; Neto and Vinson, 2011); N/A, not applicable*.

** *Concentration of a particular compound tested (added to the basal medium—0.5% agarose and 0.5% glucose)*.

To study the within-season variation of organic acids (OA) in the fruit, material for each plant-genotype was collected from 3 plots in late July (Jul 26), two times in August (Aug 6 and Aug 23), and two times in September (Sep 5 and Sep 16). After harvesting, the berries were transferred to the laboratory, immediately frozen at −80°C and stored until analyzed (within 6 months of harvest). From each sample harvested from an individual plot approximately 2 g (±1 g depending on fruit availability) of berries were randomly selected for chemical analysis.

### Effect of the tested compounds on the radial growth of selected fungal pathogens

Sixteen compounds previously reported (Table 1; Fellers and Esselen, 1955; Schmid, 1977; Coppola et al., 1978; Mäkinen and Söderling, 1980; Hong and Wrolstad, 1986; Zuo et al., 2002; Zheng and Wang, 2003; Cunningham et al., 2004; Shahidi and Naczk, 2004; Vvedenskaya et al., 2004; Singh et al., 2009; Neto and Vinson, 2011) as naturally occurring in cranberry fruit and four combinations of the green cranberry fruit tissues representing rot-resistant and rot-susceptible genotypes were incorporated into basal medium to study their effects on fungal radial colony growth. Plates (85 mm diam.) containing 20 ml of a media with test compounds (Table 1) were prepared by incorporating filter-sterilized compounds into a basal medium after autoclaving. A 10-mm plug taken from edge of an actively-growing 10-day-old culture of four selected isolates of cranberry fruit rot fungi, i.e., *Coleophoma empetri, Colletotrichum gloeosporioides, Phyllosticta vaccinii* and *P. vaccinii* growing on PDA, was used to inoculate the plates containing each of the media. The basal medium was used as a control. The plates were incubated at 22°C in darkness. The radial growth of each fungus on plates was measured 10 days after inoculation along perpendicular axes. The study was performed with three replicates for each fungus/medium combination.

### Detection of H_2_O_2_ secreted into agar by fungi

To visualize secretion and accumulation of ROS as H_2_O_2_ into agar, the plates with the 10-day-old fungal colonies were stained by flooding plates with 6 ml of 100 mM potassium phosphate buffer, pH 6.9, 2.5 mM 3,3′-diaminobenzidine tetrachloride (DAB) and 5 purpurogallin units ml^−1^ of horseradish peroxidase (Type VI-A), swirled to cover the entire surface, and incubated at room temperature for 10 h (Pick and Keisari, 1980; Munkres, 1990). To stop the color development the plates were rinsed in sterile dH_2_O. Visible ROS reaction zones were measured and each plate was photographed.

### Extraction and quantification of total benzoic acid, citric acid, malic acid, and quinic acid in developing cranberry fruit

#### Extraction of organic acids

Fruit thawed for 1 h at 22°C were homogenized using Waring™ Laboratory Blenders (Model 31BL91 with MC-1 Mini Container, Waring Commercial, Torrington, CT) prior to analyses. Homogenized fruit samples were diluted with dH_2_O in ratio approximately 1:10 and mixed by shaking. The mixture was then sonicated (Branson 3510, Branson Ultrasonics Corporation, Danbury, CT) for 10 min, transferred and left on a stirrer at 60 rpm for 10 min in a water bath (Precision 2870, Thermo Electron Corporation, Waltham, MA) with temperature of 90°C. The suspension was filtered with Whatman filter paper and 1 ml of the supernatant was centrifuged (AccuSpin Micro 17R, Fisher Scientific, Osterode, Germany) at 13,300 g for 10 min at 4°C. From each centrifuged sample 300 μl of supernatant was transferred into an HPLC vial and analyzed for organic acids.

#### High-performance liquid chromatography (HPLC) analysis

Aqueous extracts obtained from cranberry fruit were analyzed for identification and quantification of organic acids (OA) using a Dionex® HPLC system (Dionex, Sunnyvale, CA) equipped with AS50 Autosampler, AS50 Thermal Compartment, PDA-100 Photodiode Array Detector and GP40 Gradient Pump. For all standards and extracts, a Waters Atlantis dC18 column (5.0 μm particle size; 100A; 250 mm length × 4.6 mm ID; Waters Co., Milford, MA) with a security guard cartridge (Phenomenex, Inc., Torrance, CA) was used; the column temperature was 25°C. A binary solvent system with solvent A: 0.5% phosphoric acid in water and solvent B: 0.5% phosphoric acid in acetonitrile was used with isocratic elution of 0% B from 0 to 11 min; linear gradient of 0 to 20% B from 11 to 13 min; 20 to 60% B from 13 to 18 min; 60 to 80% B from 18 to 20 min; isocratic elution of 80% B from 20 to 25 min; linear gradient of 80 to 20% B from 25 to 28 min; 20 to 0% B from 28 to 30 min and isocratic elution of 0% B from 30 to 40 min at a flow rate of 0.6 ml min^−1^ with 20 μl sample injection volume.

Chromatograph peaks were identified taking into account the retention time and the UV-Vis absorption spectra of the peaks with those of corresponding standards. Photodiode Array Detector was monitored at three wavelengths, 210 nm for citric (CA) and malic (MA) acids, 214 nm for quinic acid (QA), and 230 nm for benzoic acid (BA). Data acquisition and processing were performed using Dionex Chromatography Software—Chromeleon Client version 6.80 (Dionex, Sunnyvale, CA). Quantification of all the organic acids was based on a standard curve prepared with BA, CA, MA, and QA. The contents of organic acids were expressed as milligrams of organic acid per gram of fresh weight (fw). The samples were analyzed in duplicates.

#### Calibration curves

Concentration of organic acids in cranberry ovary extractions was determined based on calibration curves. The calibration curves were constructed using the standard solutions. Known amounts of BA, CA, MA, and QA adding to double distilled water resulted in stock solutions and their serial dilutions were used to prepare standard solutions. Except for BA where 16 standard concentrations were prepared, eight standard concentrations were prepared for the other three acids and analyzed by HPLC in triplicate. Calibration curves were generated by plotting peak area (mAU) against acid concentration (mg ml^−1^) with linear regression analysis.

### Statistical analyses

Results were expressed as mean ± standard error of the mean (SEM). Significant differences (α = 0.01) between means were estimated by use of analysis of variance (ANOVA), General Linear Model (GLM) followed by the Ryan-Einot-Gabriel-Welsch Q multiple range test. The effects of plant-genotype and within-season variation were investigated with different dates analyzed independently. The data analysis was generated using SAS/STAT software, Version 9.3 of the SAS System for Windows (SAS Institute Inc., Cary, NC, USA, 2011).

## Results

### Effect of the cranberry compounds on the radial growth of selected cranberry fungi

**Figure 1:**
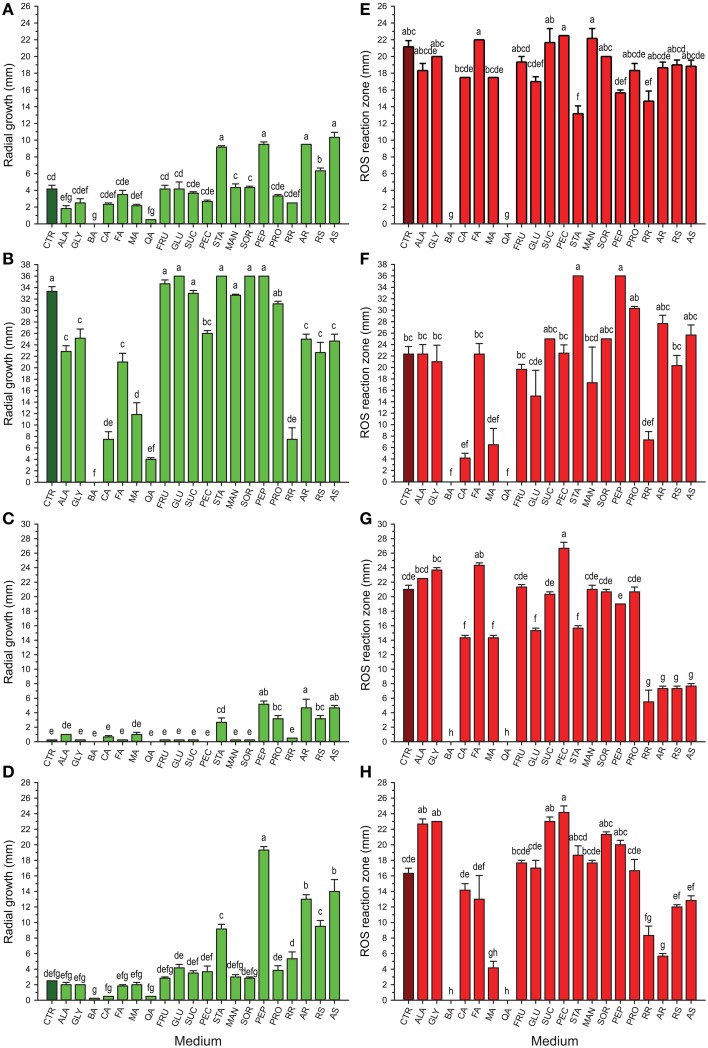
**Growth (green) of *Coleophoma empetri* (A), *Colletotrichum gloeosporioides* (B), *Phyllosticta vaccinii* (C), and *Physalospora vaccinii* (D) and secretion of hydrogen peroxide (red) into the media by *Coleophoma empetri* (E), *Colletotrichum gloeosporioides* (F), *Phyllosticta vaccinii* (G), and *Physalospora vaccinii* (H), respectively**. CTR, control (dark green or dark red); ALA, alanine; GLY, glycine; BA, benzoic acid; CA, citric acid; FA, folic acid; MA, malic acid; QA, quinic acid; FRU, fructose; GLU, glucose; SUC, sucrose; PEC, pectin; STA, starch; MAN, mannitol; SOR, sorbitol; PEP, N-Z-Soy® Peptone; PRO, Proflo Premium Quality Cottonseed protein; RR, raw rot-resistant genotype green berries; AR, autoclaved rot-resistant green berries; RS, raw rot-susceptible cranberry green berries; AS, autoclaved susceptible cranberry green berries. Values are the average of radial growth of colonies (mm; along perpendicular axes) ± standard error of the mean or reactive oxygen species (ROS) reaction zone (mm) ± standard error of the mean of hydrogen peroxide (3,3′-diaminobenzidine tetrachloride/horseradish peroxidase staining) secreted into the media in triplicates (*N* = 3). The same letters are not significantly different (*P* < 0.01; α = 0.01) as determined by the Ryan-Einot-Gabriel-Welsch Q (REGWQ) multiple range test. Organic acids (benzoic and quinic acids) show inconsistent suppression of growth but consistent suppression of ROS in all rot fungi tested. Amino acids, sugars, polysaccharides, sugar alcohols, and proteins often increase fungal growth and show no effect or increase ROS secretion by fruit rot fungi.

The results from *in vitro* screening of compounds previously identified in cranberry fruit (Table 1) on growth of selected cranberry fruit rot fungi, *Coleophoma empetri, Colletotrichum gloeosporioides, Phyllosticta vaccinii*, and *Physalospora vaccinii*, are shown in Figures 1A–D. Whereas, many of the compounds had no effect on the respective fungi, effects of some of the compounds differed greatly among the species. Starch (STA), peptone (PEP), and addition of raw susceptible (RS), autoclaved susceptible (AS), and autoclaved resistant (AR) tissue of green fruit significantly stimulated (*p* < 0.0001; α = 0.01) growth of *Coleophoma empetri, Phyllosticta vaccinii*, and *Physalospora vaccinii* (Figures 1A,C,D). Protein (PRO) also significantly stimulated (*p* < 0.0001; α = 0.01) growth of *Phyllosticta vaccinii* (Figure 1C). Alanine (ALA), benzoic acid (BA), and quinic acid (QA) significantly inhibited (*p* < 0.0001; α = 0.01) growth of *Coleophoma empetri* (Figure 1A), whereas addition of amino acids (ALA, GLY), organic acids (BA, CA, FA, MA, QA), pectin (PEC) and any kind of cranberry green fruit tissue (RR, AR, RS, and AS) in the medium significantly inhibited (*p* < 0.0001; α = 0.01) growth of *Colletotrichum gloeosporioides* compared to that of the control medium (Figure 1B) but did not inhibited other fungi.

### Detection of H_2_O_2_ secreted into culture media by fungi

**Figure 2:**
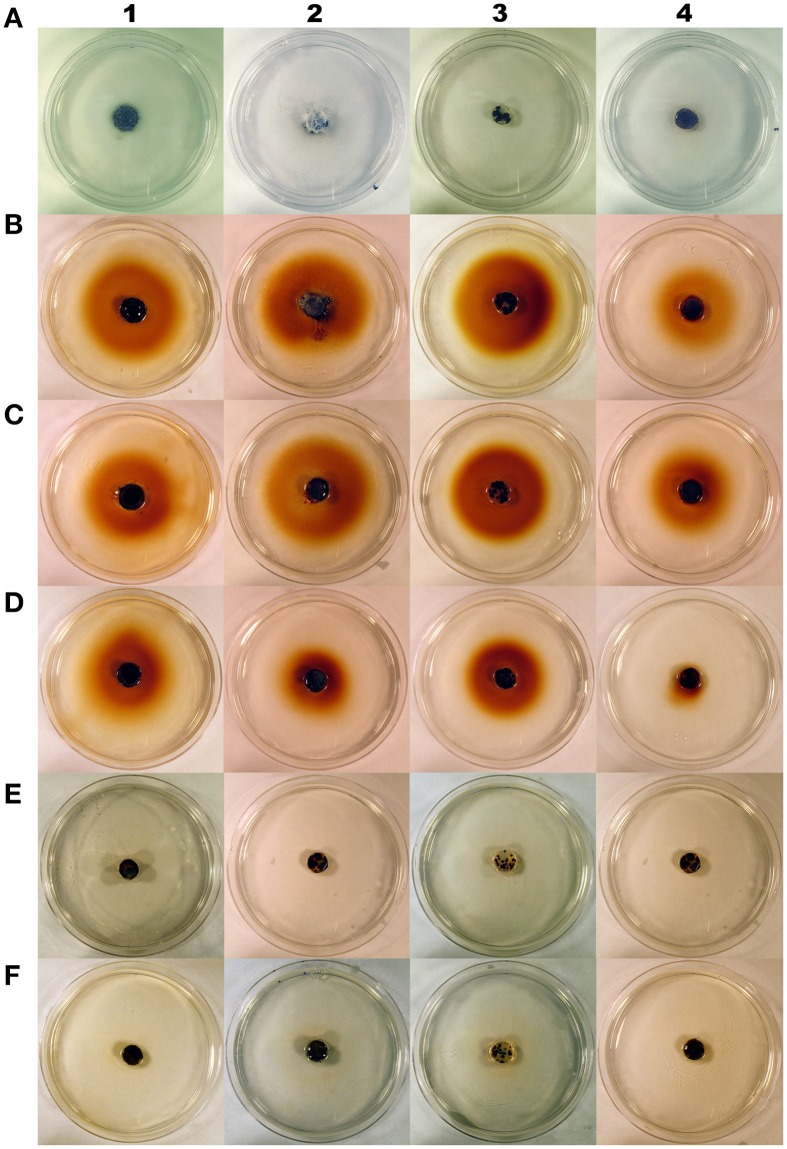
**Cranberry fruit rot fungi *Coleophoma empetri* (1), *Colletotrichum gloeosporioides* (2), *Phyllosticta vaccinii* (3), *Physalospora vaccinii* (4) grown on basal medium (0.5% agarose and 0.5% glucose; control) before staining (A), basal medium (0.5% agarose and 0.5% glucose; control) (B), and basal medium with: mannitol (C), malic acid (D), benzoic acid (E), or quinic acid (F); and then stained with 3,3′-diaminobenzidine tetrachloride/horseradish peroxidase to visualize hydrogen peroxide secretion into the media**. Red pigment around fungal colonies on stained control, mannitol and malic acid media indicates high production of hydrogen peroxide. Benzoic acid and quinic acid inhibit hydrogen peroxide production in all rot fungi tested.

Most of the compounds tested had no effects or stimulated H_2_O_2_ secretion by fungi (Table 1; Figures 1E–H, 2). However, we identified chemical compounds of cranberry fruit that inhibited H_2_O_2_ secretion by the fungi.

Addition of BA and QA into the medium completely inhibited H_2_O_2_ secretion by *Coleophoma empetri, Colletotrichum gloeosporioides, Phyllosticta vaccinii*, and *Physalospora vaccinii* (Figures 1E–H, 2). Addition of citric acid (CA) and malic acid (MA) also significantly (*p* < 0.0001; α = 0.01) reduced secretion of H_2_O_2_ into the medium by *Colletotrichum gloeosporioides* and *Phyllosticta vaccinii* (Figures 1F,G), and by *Colletotrichum gloeosporioides, Phyllosticta vaccinii*, and *Physalospora vaccinii*, respectively (Figures 1F–H, 2). However, folic acid (FA) and PEC added to the medium significantly (*p* < 0.0001; α = 0.01) stimulated production of H_2_O_2_ by *Phyllosticta vaccinii* (Figure 1G). Medium amended with glucose showed significant (*p* < 0.0001; α = 0.01) reduction of H_2_O_2_ secretion by *Phyllosticta vaccinii* (Figure 1G). Addition of both, STA and PEP to the media significantly (*p* < 0.0001; α = 0.01) reduced of H_2_O_2_ secretion by *Coleophoma empetri* (Figure 1E) but stimulated significantly (*p* < 0.0001; α = 0.01) secretion H_2_O_2_ by *Colletotrichum gloeosporioides* (Figure 1F); medium amended with STA significantly (*p* < 0.0001; α = 0.01) reduced secretion of H_2_O_2_ by and *Phyllosticta vaccinii* (Figure 1G). Media amended with ALA, glycine (GLY), sucrose (SUC), or PEC significantly stimulated (*p* < 0.0001; α = 0.01) H_2_O_2_ secretion by *Physalospora vaccinii* compared to that of the control medium (Figure 1H). Presence of RR significantly (*p* < 0.0001; α = 0.01) inhibited secretion of H_2_O_2_ into the medium by all tested fungi (Figures 1E–H); addition of RS, AR, and AS significantly (*p* < 0.0001; α = 0.01) inhibited secretion of H_2_O_2_ by *Phyllosticta vaccinii* (Figure 1G), while addition of RS significantly (*p* < 0.0001; α = 0.01) inhibited secretion of H_2_O_2_ into the medium by *Physalospora vaccinii* (Figure 1H).

### Quantification of organic acids in developing cranberry fruit

Our results showed that the levels of BA, QA, and CA in developing cranberry fruit were significantly different depending on a cranberry genotype and a fruit development stage (Table 2).

**Table 2:** **Concentrations (mg g^−1^ fresh weight) of benzoic acid (BA), citric acid (CA), malic acid (MA), and quinic acid (QA) in developing fruit of six cranberry genotypes collected during growing season**.

**Acid**	**Genotype ID**	**July 26**	**August 6**	**August 23**	**September 5**	**September 15**
BA^*^	US88-1 (R)	0.0030 ± 0.0006b	0.0107 ± 0.0017ab	0.0336 ± 0.0047a (*N* = 4)	0.0356 ± 0.0000a (*N* = 1)	0.0275 ± 0.0010b (*N* = 2)
	US88-30 (R)	0.0043 ± 0.0003ab	0.0197 ± 0.0034a	0.0483 ± 0.0020a	0.0875 ± 0.0075a	0.1816 ± 0.0096a (*N* = 2)
	US88-79 (R)	0.0091 ± 0.0016a	0.0172 ± 0.0014a	0.0405 ± 0.0063a	0.0685 ± 0.0126a	0.1221 ± 0.0126a (*N* = 4)
	US89-3 (R)	0.0037 ± 0.0004b	0.0160 ± 0.0016ab	0.0370 ± 0.0037a	0.0786 ± 0.0130a	0.1436 ± 0.0080a
	MQ (S)	0.0047 ± 0.0005ab	0.0154 ± 0.0033ab	NS	NS	NS
	ST (S)	0.0016 ± 0.0005b	0.0049 ± 0.0007b	NS	NS	NS
	Mean	0.0044 ± 0.0006	0.0140 ± 0.0010	0.0404 ± 0.0024	0.0760 ± 0.0065	0.1295 ± 0.0135
CA	US88-1 (R)	9.777 ± 0.5943a	9.240 ± 0.6713a	9.996 ± 0.2138b (*N* = 4)	20.116 ± 0.0000a (*N* = 1)	10.029 ± 0.4750a (*N* = 2)
	US88-30 (R)	10.072 ± 0.4968a	10.203 ± 0.1319a	10.442 ± 0.2651b	9.622 ± 0.3856b	10.010 ± 0.0542a (*N* = 2)
	US88-79 (R)	8.902 ± 0.2451ab	8.594 ± 0.2371a	10.305 ± 0.4255b	10.459 ± 0.7227b	8.737 ± 0.9502a (*N* = 4)
	US89-3 (R)	9.100 ± 0.4089a	9.837 ± 0.2387a	12.735 ± 0.4712a	11.025 ± 0.3531b	10.388 ± 0.2627a
	MQ (S)	6.970 ± 0.3136b	8.346 ± 0.4525a	NS	NS	NS
	ST (S)	8.560 ± 0.2478ab	9.889 ± 0.3520a	NS	NS	NS
	Mean	8.897 ± 0.2285	9.352 ± 0.1913	10.949 ± 0.2997	10.882 ± 0.5920	9.811 ± 0.3327
MA^**^	US88-1 (R)	4.974 ± 0.1741	4.605 ± 0.2750	4.721 ± 0.2871(*N* = 4)	6.585 ± 0.0000 (*N* = 1)	5.769 ± 0.5406 (*N* = 2)
	US88-30 (R)	3.744 ± 0.1216	3.916 ± 0.1346	4.419 ± 0.1341	5.193 ± 0.1530	5.347 ± 0.7554 (*N* = 2)
	US88-79 (R)	4.235 ± 0.1498	3.986 ± 0.2385	4.133 ± 0.2838	4.271 ± 0.4589	4.413 ± 0.3479 (*N* = 4)
	US89-3 (R)	3.485 ± 0.2870	4.354 ± 0.1001	4.695 ± 0.1872	4.925 ± 0.3624	5.284 ± 0.1570
	MQ (S)	5.057 ± 0.4476	5.277 ± 0.2497	NS	NS	NS
	ST (S)	3.980 ± 0.2336	4.157 ± 0.2262	NS	NS	NS
	Mean	4.246 ± 0.1421c	4.382 ± 0.1123bc	4.471 ± 0.1163bc	4.890 ± 0.2209ab	5.114 ± 0.1964a
QA	US88-1 (R)	23.161 ± 1.0241bc	22.950 ± 1.3976ab	19.810 ± 1.4333b (*N* = 4)	18.168 ± 0.0000b (*N* = 1)	14.781 ± 0.1255b (*N* = 2)
	US88-30 (R)	29.345 ± 1.2202ab	24.030 ± 1.0686ab	19.919 ± 0.5484b	18.405 ± 0.4315b	22.546 ± 4.6692a (*N* = 2)
	US88-79 (R)	21.586 ± 0.7371c	19.687 ± 0.5946b	21.379 ± 0.8786b	19.295 ± 1.0706ab	14.653 ± 0.3509b (*N* = 4)
	US89-3 (R)	31.105 ± 1.2272a	27.026 ± 0.3810a	25.469 ± 0.7081a	24.890 ± 0.7899a	21.247 ± 0.4361a
	MQ (S)	29.167 ± 2.0389ab	22.660 ± 1.5704ab	NS	NS	NS
	ST (S)	24.615 ± 1.3822abc	20.841 ± 0.9330b	NS	NS	NS
	Mean	26.496 ± 0.7824	22.866 ± 0.5672	21.811 ± 0.6426	20.722 ± 0.7908	18.625 ± 1.0899

*Results are expressed as mean ± standard error of the mean (mg g^−1^ fw); N = 6 unless indicated otherwise; MQ, Mullica Queen; ST, Stevens; R, rot-resistant genotype; S, rot-susceptible genotype; NS, no sample available due to fruit rot*.

* *Values with the same letters within a compound and within a column indicate that the genotypes are not significantly different (P < 0.01; α = 0.01) as determined by the Ryan-Einot-Gabriel-Welsch Q (REGWQ) multiple range test*.

** *No significant genotype by collection date interaction found but there was significant genotype and collection date effects (P < 0.01; α = 0.01)*.

#### Benzoic acid

**Figure 3:**
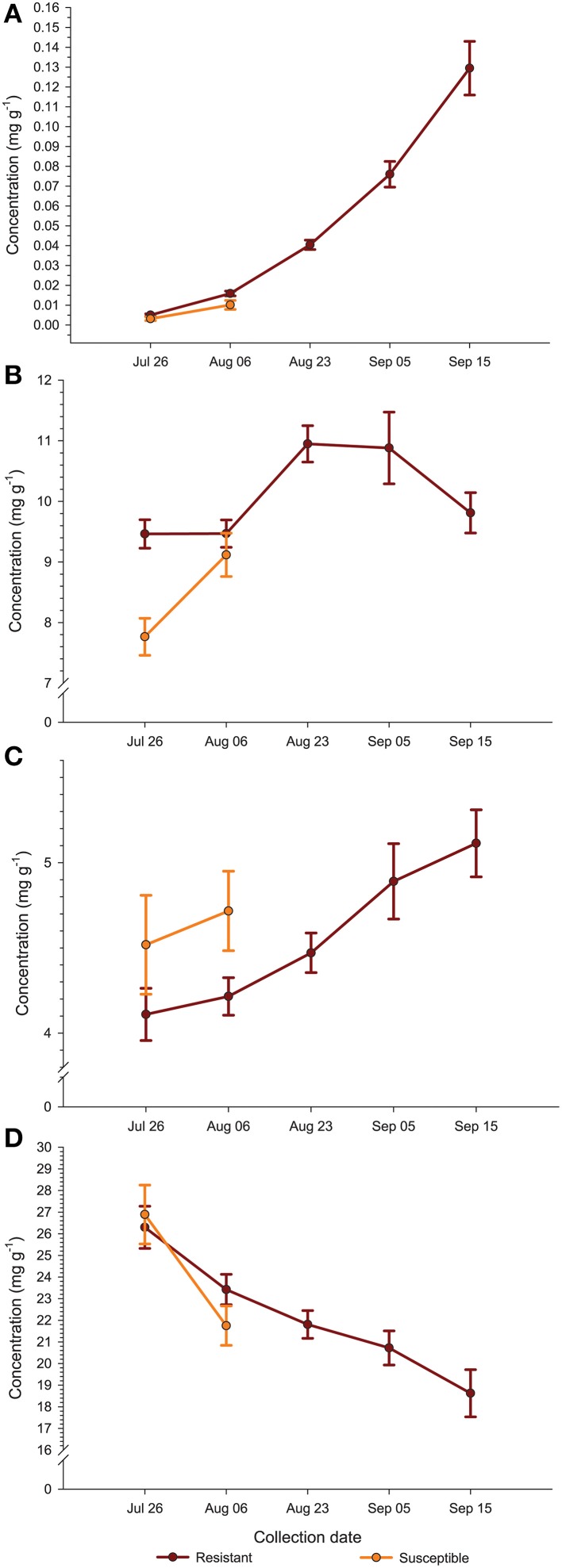
**Average (± standard error of the mean) concentration (mg g^−1^ fw) of benzoic acid (A), citric acid (B), malic acid (C), and quinic acid (D) in the fruit of rot-resistant (*N* = 4) and rot-susceptible (*N* = 2) cranberry genotypes during growing season**.

Comparing the plant genotypes within the collection date revealed a 5.7-, 4.0-, 1.4-, 2.5-, and 6-fold variation in BA content between the genotypes collected at a particular collection date (Table 2). At the first collection date (July 26), a significantly higher (*p* < 0.0003; α = 0.01) level of BA was found in the genotype US88-79 than in the US88-1, US89-3, and ST genotypes. On August 6 significantly higher (*p* < 0.0009; α = 0.01) BA content was found again in the genotype US88-79 and US88-30 than in ST. Starting with the collection date of August 23, due to severe fruit rot occurrence, there were no more fruit of the rot-susceptible genotypes (MQ and ST) available for further analysis; the number of fruit of the rot-resistant genotype US88-1 was limited as well. On both collection dates, August 23 and September 5, there was no significant variation in BA content between all analyzed rot-resistant genotypes (RR). At the last collection date (September 15), content of BA in fruit of the genotype US88-30, US88-79, and US89-3 was significantly higher (*p* < 0.0004; α = 0.01) than in the genotype US88-1. During the collection period (from July 26 to September 15), the mean level of BA in the developing fruit increased 29.4-fold, from 0.0044 to 0.1295 mg g^−1^ (Table 2; Figure 3A).

**Figure 4:**
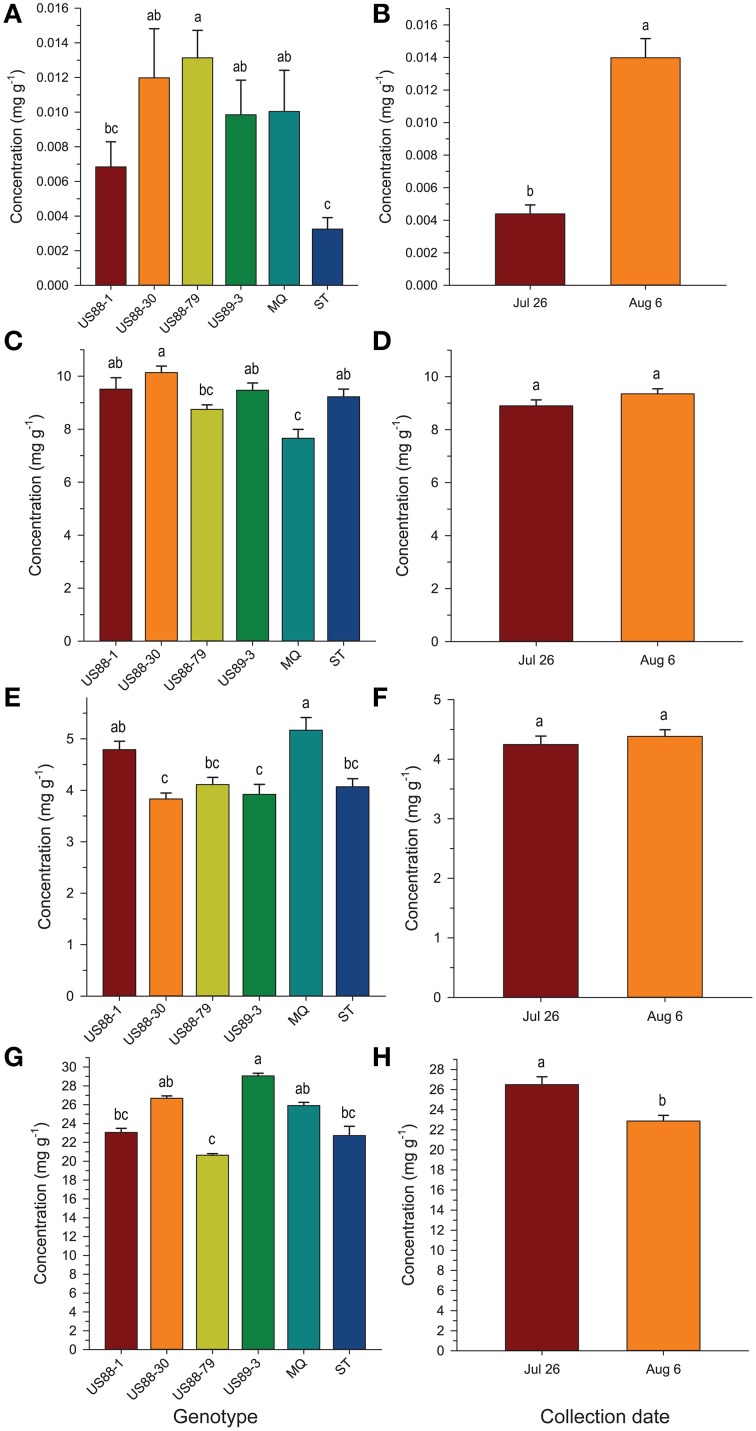
**Concentration (mg g^−1^ fresh weight) of benzoic acid (A,B), citric acid (C,D), malic acid (E,F), and quinic acid (G,H), respectively, in young fruit of six cranberry genotypes collected 2 weeks apart at the beginning of fruit development**. Values are expressed as mean ± standard error of the mean of field triplicates and laboratory duplicates (*N* = 6). Values with the same letters are not significantly different (*P* < 0.01; α = 0.01) as determined by the Ryan-Einot-Gabriel-Welsch Q (REGWQ) multiple range test.

Analysis of BA content in the first two collection dates showed significant differences between investigated genotypes and collection dates; a significantly higher (*p* < 0.0001; α = 0.01) level of BA was found in fruit of the genotype US88-79 than in fruit of ST and US88-1 genotypes (Figure 4A). Concentration of BA increased over 300% (Figure 4B) and was significantly higher (*p* < 0.0001; α = 0.01) in the second collection date (August 6) than in the first collection date (July 26).

#### Citric acid

The level of CA at the first collection date (July 26) varied and was significantly higher (*p* < 0.0001; α = 0.01) in the US88-1, US88-30, and US89-3 genotypes than in MQ (Table 2). There were no significant differences in CA content found in fruit of all analyzed genotypes at the second (August 6) and the last (September 15) collection date. On August 23 (*p* < 0.0003; α = 0.01) and September 5 (*p* < 0.0001; α = 0.01) significantly higher CA concentration was found in the genotype US89-3 and in the genotype US88-1 than in all other analyzed genotypes, respectively. However, the result obtained for the genotype US88-1 was based on one extract sample only. As the fruit developed, the mean level of CA in the cranberry fruit first increased from 8.897 mg g^−1^ on July 26 to 10.949 mg g^−1^ on August 23 and then gradually decreased to 9.811 mg g^−1^ by the end of the growing season (September 15) (Table 2; Figure 3B).

The genotype US88-30 was found to contain significantly higher (*p* < 0.0001; α = 0.01) level of CA in young fruit (the first two collection periods) than MQ and US88-79 genotypes (Figure 4C) and there were no significant differences observed between the collection dates (Figure 4D).

#### Malic acid

**Figure 5:**
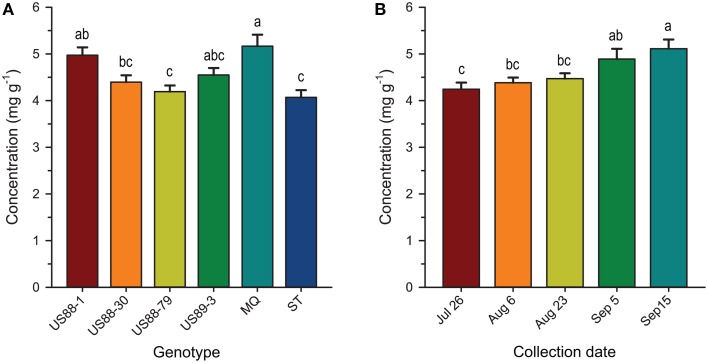
**Concentration (mg g^−1^ fresh weight) of malic acid in developing fruit of six cranberry genotypes (A) collected as the growing season progresses (B)**. Values are expressed as mean ± standard error of the mean; values for collection date represent all genotypes combined for each date; values with the same letters are not significantly different (*P* < 0.01; α = 0.01) as determined by the Ryan-Einot-Gabriel-Welsch Q (REGWQ) multiple range test.

There was no significant genotype by collection date interaction for MA content in fruit of the investigated genotypes, comparing to all collection dates (Table 2; Figure 3C). Statistical analysis revealed that during the season significantly (*p* < 0.0001; α = 0.01) higher concentration of MA was found in MQ genotype than in the genotype US88-79 and ST (Figure 5A). However, fruit of the MQ genotype, due to the severe fruit rot, were only available for analysis for the first two collection dates (July 26 and August 6). The average concentration of MA (when comparing all investigated genotypes combined for each date) increased as the season progressed (Figure 5B) and reached significantly (*p* < 0.0001; α = 0.01) higher concentration (5.114 mg g^−1^) on the final collection date (September 15).

Young fruit of the MQ genotype had a significantly (*p* < 0.0001; α = 0.01) higher MA content than the young fruit of the genotype US88-30, US89-3, US88-79, and ST while comparing to the first two collection dates (Figure 4E). However, there were no significant differences in MA content between the two collection dates found (Figure 4F).

#### Quinic acid

The content of QA in all analyzed samples varied from 14.6 to 31.1 mg g^−1^ (Table 2). There was a 1.3- to 1.5-fold variation found in the level of QA between the investigated genotypes at each collection date. On July 26, a significantly (*p* < 0.0001; α = 0.01) higher level of QA was found in the genotype US89-3 compared to US88-1 and US88-79 genotypes. At the second collection date significantly (*p* < 0.0008; α = 0.01) higher QA content was found again in the US89-3 genotype than in the genotype US88-79 and ST. A significantly (*p* < 0.0004; α = 0.01) higher level of QA was found in the genotype US89-3 on August 23 than in the other three genotypes analyzed. On September 5, the genotype US89-3 showed significantly (*p* < 0.0002; α = 0.01) higher QA content than the genotype US88-1 and US88-30. At the last collection date, content of QA was significantly (*p* < 0.0019; α = 0.01) higher in fruit of the genotype US89-3 and US88-30 than in US88-1 and US88-79 genotypes. However, genotype US88-30 was represented by two samples only. On average, the mean level of QA in fruit decreased about 30% as the fruit mature, from 26.496 mg g^−1^ on July 26 to 18.625 mg g^−1^ on September 15 (Table 2; Figure 3D).

In comparing the concentrations of QA in the young fruit during the first two collection dates, the genotype US89-3 was found to contain significantly (*p* < 0.0001; α = 0.01) higher levels of QA than fruit of genotypes US88-79, US88-1, and ST (Figure 4G). The levels of QA in fruit showed a tendency to fall between these two dates and were significantly (*p* < 0.0001; α = 0.01) lower on August 6 (Figure 4H).

## Discussion

Most of the tested cranberry compounds (Table 1) were found to increase, stimulate or only slightly inhibit H_2_O_2_ secretion, when compared to the control (Figures 1E–H). BA and QA, when added to the medium, completely inhibited H_2_O_2_ production. In addition, green fruit extract from unautoclaved cranberry fruit of RR significantly inhibited H_2_O_2_ secretion compared to that from the more rot-susceptible genotypes and/or the control (Figures 1E–H). Autoclaving of fruit resulted in loss of inhibitory activity; autoclaving may have caused denaturation or degradation of QA and other inhibitors.

Our data show that levels of QA in cranberries of the six tested genotypes decreased throughout fruit development (Table 2) and the RR showed a more gradual decline in QA levels during the first 2 weeks of our study than that observed in the more rot-susceptible genotypes (Figure 3D). At the same time, the content of BA in fruit increased as the fruit developed. In general, more RR showed higher levels of BA early in fruit development, i.e., in the first 2 weeks of our study (Table 2; Figure 3A). The levels and trends for BA and QA we found to be consistent in two growing seasons (unpublished data). Chemical and physical factors are thought to account for differences often observed in disease resistance among different fruit development stages (Prusky, 1996). Kalt and McDonald (1996) found that contents of CA and QA in Lowbush blueberry (*Vaccinium angustifolium* Aiton) were lower in overripe compared to underripe berries, while the concentration of MA was at similar levels among all maturity groups. These authors (Kalt and McDonald, 1996) also reported significant cultivar differences in CA, QA, but not MA. Observed decreases in content of QA and increases in BA levels in cranberry fruit as the season progresses may be related to increasing levels of fruit infection by fungi (Tadych et al., 2012). The high content of QA in cranberry fruit might indicate its importance as a chemical defense compound (Grayer and Kokubun, 2001) and perhaps also as a precursor for antimicrobial secondary metabolites (Weinstein et al., 1961; Boudet, 1980; Hawkins et al., 1993; Richards et al., 2006; Pero et al., 2008; Dewick, 2011; Tzin et al., 2012; Ghosh et al., 2014).

### Mechanism of action

Our experiments were focused on understanding the effects of cranberry compounds on pathogenicity behavior (e.g., ROS secretion) of the cranberry fungi. However, earlier research has associated H_2_O_2_ and other ROS secretion by fungal necrotrophs with the initial trigger of localized cell death and necrosis of host tissues (Govrin and Levine, 2000). Prevention of ROS secretion by fungi may prevent the initiation of the hypersensitivity response in fruit tissues. The likely mechanism of action for the ROS suppression effect of BA and QA is through inhibition of oxidase and oxygenase enzymes that are responsible for production of reactive oxygen. Among the oxidase enzymes of fungi are laccases that play roles in pathogenesis, detoxification, polyphenolic, and lignin degradation (Claus, 2004; Solomon et al., 2014). Generalized inhibitory activity to oxidase enzymes suggests the potential that plant produced ROS-suppressive compounds like BA may also inhibit oxidases in plants. BA is well documented to have a direct inhibitory effect on the cyclooxygenases (COX) of animals (Marnett and Kalgutkar, 2004; Corazzi et al., 2005). One of the known cyclooxygenases is COX-2, an inducible isoform of cyclooxygenase enzyme responsible for the production of pro-inflammatory prostaglandins in inflamed and neoplastic tissues. In animals the inhibition of the COX-2 enzyme by BA results in reduced ROS production with a consequent reduction in inflammation. Because BA has this effect, it is generally considered to be an anti-inflammatory. Among plant oxygenases are pathogen-induced oxygenases (PIOX) (Sanz et al., 1998). PIOX enzymes produce ROS defensively in response to invasion by pathogens. The PIOX enzymes of plants have been shown to have considerable homology to COX-2 in animals (Jahabbakhsh-Godehkahriz et al., 2013). Because of these similarities, it seems likely that oxygenase inhibitors, like BA and QA, may have ROS suppressive effects in both fungus and host tissues, essentially suppressing both fungal and plant secretion of ROS and preventing the hypersensitive response in the host.

### Growth inhibitory action of benzoic acid and quinic acid

Benzoic acid naturally occurs in both plant and animal tissues with low or no toxicity evident. However, it is known to inhibit some bacteria and fungi by reducing respiration (Warth, 1991). Because of this effect, BA and its salts, calcium benzoate, potassium benzoate, and sodium benzoate, are used at levels ranging from 0.03 to 0.3% as preservatives in food products, beverages, dentifrices, cosmetics, and pharmaceuticals to prevent decomposition by microbial growth (Krebs et al., 1983; FAO/WHO Food Standards, 2013).

Benzoic acid is known as one of the simplest of phytoalexins (Harborne, 1983; Grayer and Kokubun, 2001). The resistance of immature apples to *Neonectria ditissima* (Tul. & C. Tul.) Samuels & Rossman (syn. *Nectria galligena* Bres.) is thought to be related to the presence of BA in apples, which is accumulated by the apple after fungal infection (Brown and Swinburne, 1971, 1973; Seng et al., 1985). Exogenous applications of BA *in vitro* (at 9 mM concentration) completely inhibited the growth of *Bipolaris oryzae* (Breda de Haan) Shoemaker (the casual agent of rice brown spot disease) and under field conditions (at 20 mM), significantly reduced both disease severity and incidence in plant leaves as well as led to a significant increase in grain yield (Shabana et al., 2008). Similarly, BA at a concentration of 20 mM significantly reduced growth and spore germination of *Fusarium oxysporum* Schlect. emend. Snyd & Hans, *Fusarium solani* (Mart.) Sacc. and *Rhizoctonia solani* Khun (Shahda, 2000). Diversity and successional changes in populations of fungi in cranberry fruits were observed as the fruits developed and the season progressed (Tadych et al., 2012). The fungi possibly stimulate biochemical responses in the fruit leading to synthesis of BA in the fruit. To the best of our knowledge the role of BA as a phytoalexin in cranberry was never documented, although its function as an antifungal compound is well known. As this study shows, young, green cranberry fruits did not accumulate BA, but its content gradually increased as the season progressed.

Özçelik et al. (2011) found that QA may also act as an antimicrobial agent. However, other studies were contrary and indicated that QA alone did not have inhibitory effects, or even stimulated growth of various microorganisms (Clague and Fellers, 1934; Valle, 1957; Sokolova, 1963; Kallio et al., 1985; Bartz et al., 2013). Growth rate of an organism is not necessarily an indication of its pathogenicity and virulence. Although QA stimulated growth of *Rhizoctonia solani*, it significantly reduced fungal production of plant growth regulators belonging to phenylacetic acid metabolic complex, and as a result, suppressed disease development on tomato plants (Bartz et al., 2013). In our study, QA and BA added to the medium did not entirely suppress growth of the fruit rot fungi, consistent with previous observations, but what might be more significant for fruit rot disease development, they completely inhibited H_2_O_2_ production and its secretion into the medium (Figures 1, 2E,F) and may have inhibited secretion of other virulence factors.

## Conclusions

Based on our studies, further examination of organic acids for their virulence inhibition effects seems warranted. To reduce plant disease it may be a viable strategy to select crop plants that maintain higher levels of organic acids or other potential virulence suppressors through plant development. We propose that organic acids and other compounds should be examined as potential modulators of virulence in fungi and defensive reaction in hosts.

### Conflict of interest statement

The authors declare that the research was conducted in the absence of any commercial or financial relationships that could be construed as a potential conflict of interest.
